# Gastric Mucosal Changes Caused by Lugol's Iodine Solution Spray: Endoscopic Features of 64 Cases on Screening Esophagogastroduodenoscopy

**DOI:** 10.1155/2010/494195

**Published:** 2010-04-12

**Authors:** Daisuke Tsurumaru, Takashi Utsunomiya, Shuji Matsuura, Masahiro Komori, Satoshi Kawanami, Tatsuyuki Ishibashi, Hiroshi Honda

**Affiliations:** Departments of Clinical Radiology, Graduate School of Medical Sciences, Kyushu University, 3-1-1 Maidashi, 812-8582 Higashi-ku Fukuoka City, Japan

## Abstract

*Aim*. To clarify the endoscopic mucosal change of the stomach caused by Lugol's iodine solution spray on screening esophagogastroduodenoscopy (EGD). *Methods*. Sixty-four consecutive patients who underwent EGD for esophageal squamous cell carcinoma screening were included in this study. The records for these patients included gastric mucosa findings before and after Lugol's iodine solution was sprayed. The endoscopic findings of the greater curvature of the gastric body were retrospectively analyzed based on the following findings: fold thickening, exudates, ulcers, and hemorrhage. *Results*. Mucosal changes occurred after Lugol's solution spray totally in 51 patients (80%). Fold thickening was observed in all 51 patients (80%), and a reticular pattern of white lines was found on the surface of the thickened gastric folds found in 28 of the patients (44%). Exudates were observed in 6 patients (9%). *Conclusion*. The gastric mucosa could be affected by Lugol's iodine; the most frequent endoscopic finding of this effect is gastric fold thickening, which should not be misdiagnosed as a severe gastric disease.

## 1. Introduction

It is widely accepted that chromoendoscopy using Lugol's iodine solution is effective for the detection of esophageal squamous cell carcinomas. Mature squamous epithelia, which contain glycogen, are stained deeply by iodine, while mucosal lesions such as dysplasias or carcinomas, which contain little or no glycogen, remain unstained in contrast to the surrounding mucosa [1–4]. Lugol's iodine, however, can induce mucosal irritation, leading to retrosternal pain and discomfort, and can even induce erosion or ulceration in the esophagus and stomach [1, 5, 6]. There have been several case reports of esophageal and gastric injury caused by Lugol's iodine [5–7]. In spite of these facts, actual mucosal changes or damage caused by Lugol's iodine solution have not been sufficiently documented and remain unclear.

The aim of our study was to clarify the endoscopic mucosal change of the stomach caused by Lugol's iodine solution spray on screening esophagogastroduodenoscopy (EGD).

## 2. Materials and Methods

### 2.1. Patients

Sixty-four consecutive patients (54 men and 10 women; age range, 36–80 years; mean age 64 years) who underwent EGD for esophageal squamous cell carcinoma screening during the period from March 2007 to March 2008 at the Department of Clinical Radiology, Kyushu University Hospital, Fukuoka, Japan, were included in this study. The records for these patients included gastric mucosa findings before and after Lugol's iodine solution was sprayed. The patients' profiles are shown in Table 1. Written informed consent was obtained from all patients before endoscopic examination. Patients with stomach illness such as gastric malignancies, rugae hypertrophy, ulcers, postoperative stomach, and acute gastritis were excluded from this study.

### 2.2. Endoscopic Examination

The endoscopies were performed by three experienced endoscopists using standard endoscopes (GIF-KQ240, GIF-Q260, or GIF-H260; Olympus Optical Co, Ltd, Tokyo, Japan). Patients were given premedication with local pharyngeal anesthesia and diazepam (5–10 mg intravenously) if needed and were placed in the left lateral decubitus position. The endoscopic procedure was as follows:

1. routine examination of the esophagus, stomach, and proximal duodenum,
2. examination of the esophagus using 10 mL of 3% Lugol's iodine solution (6 g of I and 12 g of KI in 500 mL water) spray administered by a hand-controlled syringe via the working channel of the endoscope over the entire esophagus, followed by the spraying of 20 mL of 20% sodium thiosulfate solution (STS; 10% Detoxol, Banyu Pharmaceutical, Co., LTD., Tokyo, Japan) in order to neutralize the Lugol's iodine solution,
3. aspiration of the residual agent of the stomach via the working channel, followed by reexamination of the stomach under the same conditions as before the Lugol's solution spray was administered. The time interval between Lugol's iodine spray and reexamination was 64–970 (mean; 174) seconds.

Endoscopic examination records and their pictures were retrospectively analyzed. The endoscopic findings of the greater curvature of the gastric body where the agent had collected were evaluated based on the following findings: fold thickening, exudates, ulcers, and hemorrhage. The association of the mucosal changes with the time that Lugol's solution was in contact with the gastric mucosa was also analyzed using Student's *t*-test. Endoscopic biopsy specimens, if associated with the mucosal changes, were reviewed for histological confirmation.

## 3. Results and Discussion

### 3.1. Results

**Figure 1:**
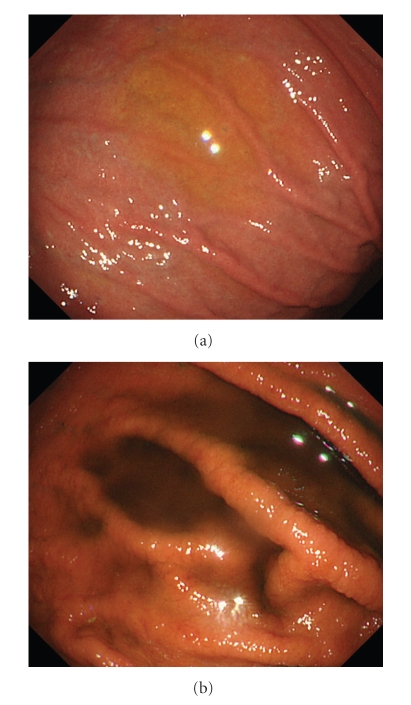
Gastric mucosal changes occurred after splaying Lugol's iodine solution in a 78-year-old man. (a) Endoscopic view of the greater curvature of the gastric body showed no fold thickening. (b) Endoscopic view after spraying Lugol's iodine solution showed fold thickening.

**Figure 2:**
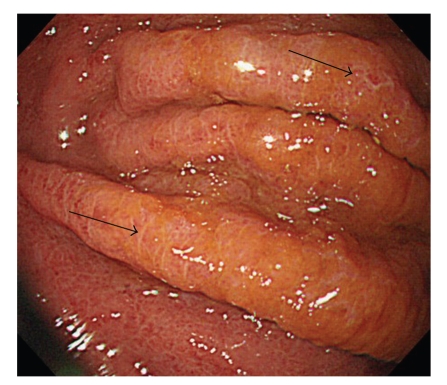
Gastric mucosal changes caused by Lugol's solution in a 58-year-old man. The reticular pattern of white lines on the thickened folds is shown by the arrows.

Mucosal changes of the greater curvature of the gastric body occurred after Lugol's solution spray totally in 51 patients (80%). Fold thickening was observed in all 51 patients (80%), and a reticular pattern of white lines was found on the surface of the thickened gastric folds in 28 of the patients (44%). Exudates were observed in 6 patients (9%). No ulcers, hemorrhage, or other complications occurred during the endoscopic examination in this study (Table 2). The characteristic endoscopic findings are shown in Figures 1 and 2. The mean time intervals between Lugol's solution spray and reexamination were 166 ± 156 seconds for positive mucosal changes group and 206 ± 248 seconds for negative group, which presented no significant difference (*P* = .109). No biopsy specimens were obtained from the stomach after spraying with Lugol's solution. Esophageal biopsies were performed in 8 patients for lesions that were suspicious of malignancy after spraying Lugol's solution, but no pathological lesions that could be attributed to the agent were found. There were no other unusual esophageal lesions found during the endoscopic procedures, and there were no esophageal or gastric adverse events identified clinically within 48 hours of any of the cases.

### 3.2. Discussion

Drug-induced gastritis, although rare, is known as an etiology of noninfective gastritis. Numerous drugs including iron, colchicine, kayexalate in sorbitol and various chemotherapeutic agents have been associated with gastric mucosal changes [8]. However, Lugol's iodine solution has not been generally recognized as potentially toxic for the gastric mucosa. Free iodine can cause mucosal irritation leading to retrosternal pain and discomfort and can even result in erosions or ulcers in the esophagus and/or the stomach [1]. There are several case reports of esophageal and gastric injury caused by Lugol's iodine [5–7]. 

In our study, endoscopically evident gastric mucosal changes appeared after the spraying of Lugol's iodine solution in 80% of the patients, consistent with a direct effect of Lugol's iodine on the gastric mucosa. Thickened gastric folds were the most frequent mucosal change seen. Sreedharan et al. reported a case of gastric mucosal damage during Lugol's chemoendoscopy which showed a similar endoscopic appearance, and their biopsy specimens showed acute edema of the gastric lamina propria with loss of the superficial epithelium but no inflammatory infiltrate, consistent with an acute toxic gastric mucosal injury induced by Lugol's iodine solution [7]. They found these changes only in the greater curvature of the gastric body, where the Lugol's solution pools during the EGD exam. We analyzed the mucosal changes caused by Lugol's solution in the greater curvature of the gastric body on the basis of their results. We did not have endoscopic data from other parts of the stomach. The endoscopic appearance of the esophagus showed no abnormalities that could be attributed to the spraying of Lugol's solution. These results may indicate that, as Sreedharan et al. proposed, the gastric columnar epithelium may be more susceptible to the toxic effect of Lugol's iodine than the squamous esophageal mucosa. Another patient, reported by Park et al., had much more severe esophageal and gastric injury after Lugol's spraying, and they hypothesized that this extreme damage might have been caused by a hypersensitivity reaction [5]. However, our results indicated that the mucosal changes that appeared after Lugol's solution spray were not associated with a hypersensitivity reaction because of our negative esophageal findings.

Sreedharan et al. suggested aspirating the gastric pool as soon as possible after spraying with Lugol's, before examining the esophagus, to reduce the toxic effects. However, we found no significant difference between the incidence of gastric mucosal changes and the exposure time of Lugol's solution in our study.

Fold thickening is well known as one of the endoscopic findings of major gastric disease. The causes of gastric fold thickening are protein-losing gastropathy with hypertrophic gastric folds (PLGH) including Menetrier's disease, anisakiasis, acute gastric mucosal lesions, gastric lymphoma, and scirrhous carcinoma [9, 10]. Acute gastric fold thickening induced by Lugol's iodine solution should be recognized as a minor iatrogenic disorder that might be confused with these more serious conditions.

A reticular pattern of white lines on the swollen gastric folds was seen in 44% of the patients. This may have indicated severe interstitial edema of the columnar epithelium, because it was most frequently seen in the more thickened folds. However, it must remain a hypothesis and cannot be tested because endoscopic measuring or pathological correlation was not available in this retrospective study. 

Kondo et al. reported that sodium thiosulphate solution spray (STS) neutralized free iodine and reduced the symptoms induced by Lugol's iodine [1]. In the present study, the gastric mucosal change might be somewhat affected by STS because endoscopic evaluation was performed after STS spraying. Therefore, both endoscopic and pathological investigations without STS are needed in order to verify the actual effects of Lugol's iodine on the gastric mucosa.

Our study has two limitations mentioned above. First, the pathological correlation was absent. Second, accurate evaluation of swollen gastric folds was difficult because it would be influenced by gas distention.

We might have to consider designing a prospective study to collect the data needed to further evaluate the causes of the post-Lugol's changes and to propose the prevention strategies.

## 4. Conclusions

In conclusion, it is important to be aware that the gastric mucosa could be affected by Lugol's iodine, and that the most frequent endoscopic finding of this effect is gastric fold thickening. Therefore, endoscopists should take into consideration the possibility of this adverse effect and examine the stomach before spraying the agent to avoid misdiagnosing the thickened fold as a severe gastric disease upon screening EGD.

## Figures and Tables

**Table 1:** The clinical characteristics of the patients (*n* = 64).

Age, mean *y* (SD)	64 (10)
Sex, male, *n* (%)	54 (84)
Reason for screening endoscopy, *n* (%)	
Previous malignancy	
Esophageal cancer	23 (36)
Pharyngeal cancer	18 (28)
Tongue cancer	9 (14)
Laryngeal cancer	4 (6)
Oral cavity cancer	3 (5)
Other	7 (11)

**Table 2:** Endoscopic findings of the greater curvature of the gastric body after spraying Lugol's iodine solution.

Endoscopic findings (*n* = 64)	*n* (%)
Thickened folds	51 (80)
Reticular pattern	28 (44)
Exudates	6 (9)
Ulcers	0 (0)
Hemorrhage	0 (0)
